# Mesenchymal stem cell–gut microbiota interaction in the repair of inflammatory bowel disease: an enhanced therapeutic effect

**DOI:** 10.1186/s40169-019-0251-8

**Published:** 2019-12-23

**Authors:** Dickson Kofi Wiredu Ocansey, Li Wang, Jingyan Wang, Yongmin Yan, Hui Qian, Xu Zhang, Wenrong Xu, Fei Mao

**Affiliations:** 1 Key Laboratory of Medical Science and Laboratory Medicine of Jiangsu Province, School of Medicine, Jiangsu University, 301 Xuefu Road, Zhenjiang, 212013 Jiangsu People’s Republic of China; 2 Huai’an Maternity and Children Hospital, Huaian, 223002 Jiangsu People’s Republic of China; 3 Directorate of University Health Services, University of Cape Coast, Cape Coast, Ghana

**Keywords:** Mesenchymal stem cell therapy, Fecal microbiota transplant, Inflammatory bowel disease, MSC–gut bacteria interaction, Combined therapy

## Abstract

**Background:**

Several investigations affirm that, patients with inflammatory bowel disease (IBD) exhibit dysbiosis characterized by restricted biodiversity and imbalanced bacterial composition intertwined with immune dysregulation. The interaction between stem cells and gut microbiota is a novel and highly promising field that could add up to a better understanding of the gut physiology, as well as therapeutic improvement towards diseases like IBD. Through direct contact or release of products and/or metabolites, gut bacteria regulate gut homeostasis, damage repair, regeneration and differentiation of stem cells. In the same way, mesenchymal stem cells (MSCs) produce similar effects including restoration of gut–microbiome composition.

**Body:**

We reviewed the anti-inflammatory, antimicrobial, pathogenic bacterial clearance, proliferation and tissue remodeling effects of mesenchymal stem cells (MSCs) and fecal microbiota transplantation (FMT) as separate transplants in IBD, and the outcome of the interaction between MSCs and gut microbiota.

**Conclusion:**

The two therapies share several points of connection in therapeutics with enhanced functionalities in their interaction with each other. Focused investigations of MSC–gut bacteria interactions could lead to a novel discovery in therapeutics. We also anticipate an improved clinical remission rate in a combined FMT–MSC transplantation approach in IBD than the current single FMT or MSC approach.

## Background

Microbiome is known to partake in a wide range of important roles such as hematopoiesis [1], immune system modulation and development [2], neurologic signaling [3], host metabolism [4], and remodeling of bone mass [5] in the mammalian tissue. Characterized by chronic and relapsing intestinal mucosa inflammation, inflammatory bowel disease (IBD) is generally defined as either Crohn’s disease (CD) or ulcerative colitis (UC) with related causes being genetic, gut (microbial and immune changes), environmental and lifestyle factors. The participation of gut–microbiota in the pathophysiology of IBD has lately been highlighted with the outcome suggesting a crucial function of the gut–microbiota in the intestinal inflammation and even in colorectal cancer [6]. Even though it has not clearly been determined how the dysbiosis observed participates in intestinal inflammation, it is however recognized that several of the documented IBD susceptibility genes are linked with recognizing and processing bacterial cells, which agrees with certain function of the gut–microbiota in IBD pathogenesis. On the basis of this, some therapeutic experimental models and clinical trials that seek to correct the alterations within the gut–microbiota which include fecal microbiota transplantation and probiotics administration have shown promising outcomes in IBD [7].

Transplanted MSCs have also shown significant contributions towards the recovery of many diseases including IBD via engraftment and differentiation into functional reparative cells, replacement of injured tissues as well as the use of immune modulators or trophic resources. These mostly result in expressed paracrine factors that ultimately promote tissue repair [8]. MSC therapy has also been demonstrated to dampen inflammation, restore gut microbiome alteration and enhance pathogenic bacterial eradiation culminating in reestablished gut health in IBD. Even though little is known about MSC–gut microbiota interaction, the few available studies indicate a positive communication which results in enhanced functions of both components. In this document, we review the therapeutic application of gut microbiota (FMT) and MSC in IBD, emphasizing on the common characteristic effects of these two therapies and how their interactions potentiate the functions of each other. We also discuss the way forward to a possible future FMT–MSC combined therapeutic approach.

## Features that differentiate UC and CD

Regardless of the fact that both UC and CD are labelled as IBD, there are significant differences between the two. These differences invariably affect their pathology and response to therapy. In CD, the location of the inflammation may occur anywhere along the digestive tract with deeper ulceration, thickened colon wall, patched inflammatory pattern, granulomas and possible fistulas, strictures and fissures in the complicated state [9]. On the other hand, UC is typically restricted to the large intestine (colon) with surface mucus lining ulceration, thinner and continuous inflammation of the colon wall with no patches, no granulomas, fistulas, strictures and fissures [9, 10].

A recent report indicates that at the pathway level, virus infection and autoimmune pathways are upregulated in CD but not in UC whilst pattern recognition-mediated innate immune pathways (TLR2 and TLR4) are appreciably raised in UC but not in CD [11]. This report identifies different intervention targets for effective treatment of the two diseases. The microRNA signatures of body fluids and tissues like saliva, blood and colon have also indicated significant variations between UC and CD. Schaefer et al. documented that, about 26 miRNAs are changed in UC and CD colon biopsies relative to non-IBD controls. Out of this number, UC was associated with the differential expression of 6 miRNAs whilst CD was associated with 10 miRNAs in matched colon tissues. In whole blood, altered expression of 9 miRNAs were linked to UC whilst 6 miRNAs were linked to CD. Similar alterations in expression were also noticed in the saliva of UC and CD patients [12]. This aberrant miRNA expression profiles are believed to contribute the IBD pathogenesis. Other approaches that have been employed to reveal the differences between these two diseases include signaling pathways and gene expressions [13], specific inflammasome [14] and extracellular matrix turnover profile [15].

## Role of gut microbiota in IBD

Researches sprouting out within the cross point between IBD and the microbiota are very promising and believed to soon significantly impact daily medical practice. Microbial profiles sometimes called “signatures” vary appreciably and enough between the diseased and the healthy individual [16]. The gut–microbiota has physiological functions that provide several health imparts to the host in relation to nutrition, pathogen protection, metabolism and immunity [17]. However, recent advances in clinical and experimental research have discovered alterations in the function and composition of the gut–microbiota (dysbiosis) in several diseases including IBD [18]. Though the exact cause of IBD is still not known, it is documented that its pathogenesis is closely linked with dysbiosis with the most consistent observation being reduced bacterial diversity; a decrease of *Firmicutes*, and an increase of *Proteobacteria* [7].

Research has confirmed several specific role of certain gut bacteria in relation to IBD pathogenesis and recovery. In some of these investigations, a reduction in *Firmicutes* such as *F. prausnitzii, Roseburia inulinivorans, Blautia faecis, Clostridium lavalense* and *Ruminococcus torques* were noticed in persons having CD compared to the healthy individuals [19, 20]. The quantity of *F. prausnitzii* in the gut correlated significantly with risk of ileac CD relapse after surgery and its population reconstitution after relapse is linked with the maintenance of clinical remission. Again, a decrease of *Roseburia* spp. predisposes a healthy individual to a high genetic risk for IBD [21]. However, an increase in *Proteobacteria,* mainly *E. coli* (38%), was observed in active CD patients compared to only 6% in healthy people [22]. Other increased bacteria population associated with IBD include mucolytic bacteria *Ruminococcus torques* and *Runinococcus gnavas* [23]. These gut bacteria adhere to the intestinal epithelium consequently affecting intestinal permeability, altering diversity and composition of gut–microbiota and triggering inflammatory responses through the regulation of inflammatory genes expression leading to intestinal inflammation [24].

**Fig. 1:**
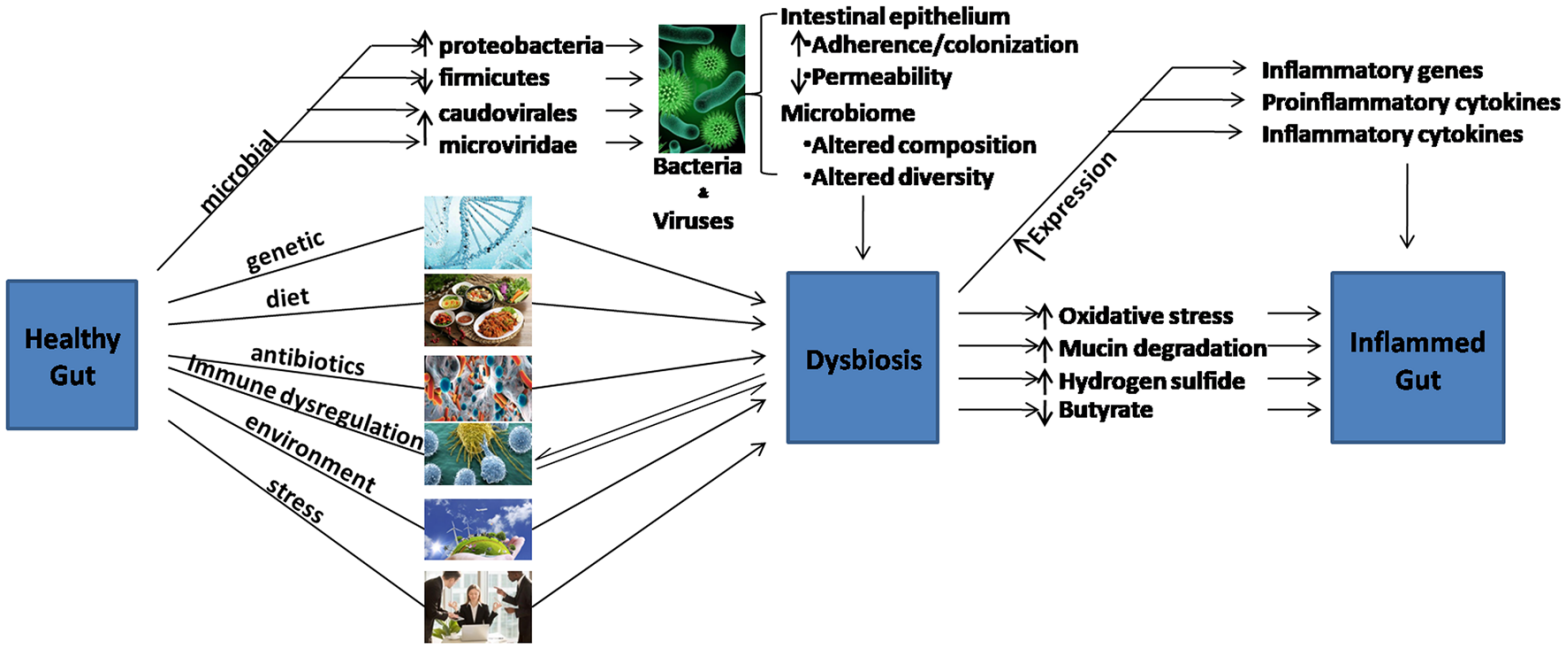
The road of a healthy gut to an inflamed gut. Many factors are associated with the alteration of gut microbiome which ultimately lead to dysbiosis. The altered microbiota diversity and composition coupled with other intestinal epithelial changes lead to inflammation in the gut, characterized by increased inflammatory genes expression

Aside bacteria, the gut microbiota comprise of other microorganisms including fungi and viruses which are possible key factors in bacterial population control and even direct participation in the pathogenesis of IBD [16]. With regards to specific distortions of enteric virome in IBD, it is known that bacteriophages of the *Caudovirales* and *Microviridae* families are the most abundant enteric virome within the healthy populace. In IBD patients however, these bacteriophages richness especially the *Caudovirales* family are increased compared to the healthy individual [25]. Several other factors link a healthy gut to dysbiosis and consequently, inflammatory gut (Fig. 1).

## Fecal microbiota transplantation in IBD

IBD is believed to exhibit a complex interplay of dysbiosis and dysregulation of the immune system [26]. Evolving therapies that seek to correct these abnormalities include the growing interest to rectify the underlying dysbiosis via the application of FMT. While this therapy has indicated great efficacy in refractory *Clostridium difficile* infections, its general application is yet to be definitely proven in IBD [27]. In one study, FMT administered once weekly via enema for 6 weeks was superior to placebo with some differences in efficacy in relation to donors [28]. In a similar study, no noteworthy variation in endoscopic and clinical remission was witnessed between participants who received their own gut microbiome and those who received from donors when the FMT was administered through nasoduodenal tube [29]. This raises concerns on donor selection, route and frequency of administration, FMT preparation and receiver factors amongst others in the quest to obtaining optimum therapeutic influence. There is therefore the need for specifically designed experiments in and around these optimal factors in FMT application. An earlier research that investigated FMT treatment efficacy in IBD documented an overall clinical remission rate of 45% (54/119) in patients during follow-up and concluded that, FMT is safe but has variable efficacious outcome in IBD treatment [26]. Furthermore, a recent systemic review and metaanalysis also asserted that, in spite of the small number of identified studies and the low quality of evidence, FMT is promising and capable of increasing the percentage of clients that achieve clinical remission in UC [30]. Some of these clinical trials are presented in Table 1.

**Table 1:** Samples of documented application of FMT in IBD clinical trials

Type of IBD	Study design/aim	Volume/frequency	Route	Observed outcome	References
UC	Efficacy evaluation	24 g/250 ml 20 g/100 ml 3 days	Nasojejunal tube Enema	1/5 (20%) had clinical response with effective augmentation by FMT Side effects included temporal rise in C-reactive protein and fever	[31]
UC	Prospective and uncontrolled study	250 ml 5 rounds	Duodenal gastroscopy	Significantly reduced clinical index scores for diarrhea, abdominal pain, blood stool and intestinal mucosal lesions No serious adverse reactions	[32]
CD with inflammatory mass	Efficacy and safety evaluation	Repeated every 3 months after initial dose	Mid-gut Transendoscopic enteral tubing	68% (17/25) and 52% (13/25) clinical response and clinical remission at 3 months respectively At 6, 12 and 18 months, clinical remission were 48% (12/25), 32% (8/25) and 22.7% (5/22), respectively No severe adverse events	[33]
UC	Pilot study on feasibility and safety	165 ml/day 5 days	Enema	7/9 (78%) had clinical response within 1 week 6/9 (67%) maintained clinical response at 1 month No serious adverse event	[34]
UC	Randomized controlled trial	Days 1 and 21	Nasoduodenal tube	7/23 (30.4%) had clinical remission in intention-to-treat analysis 7/17 (41.2%) had clinical remission in the per-protocol analysis 2 patients had FMT-linked adverse events Responders had similar microbiota as that of donors by 12 weeks	[29]
Fistulizing CD	A case study	150 ml once	Mid-gut gastroscopy	Significantly alleviated fever, improved bloody purulent stool and decreased abdominal pain, with reduced intraperitoneal inflammatory mass at 1 week Clinical remission at 1 month Sustained clinical remission with resolved mass without exudation at 3 months	[35]
Refractory CD	Pilot study on feasibility, efficacy and safety	Once	Mid-gut	86.7% (26/30) and 76.7% (23/30) had clinical improvement and remission respectively at 1 month	[36]
Mild to severe CD	Evaluation of efficacy in the short term and risk factors in the long term	184 frequencies	Mid-gut	Clinical response and clinical remission were 45% (9/20) and 20% (4/20) in patients with adverse events, and 75.6% (90/119) and 63.0% (75/119) in patients without adverse events respectively Adverse events of 21.7% in manually prepared FMT and 8.7% in automated preparations Manual or automatic purification of fecal microbiota had no correlation with the efficacy of FMT	[37]
UC	Randomised placebo-controlled trial	5 days per week for 8 weeks	Colonoscopy Enemas	11/41 (27%) who received FMT as against 3/40 (8%) who received placebo had steroid-free clinical remission with endoscopic remission or response Adverse events recorded in 32/41 (78%) FMT and 33/40 (83%) placebo patients with serious events in 2 FMT and 1 placebo patients	[38]
CD	Prospective open-label study (uncontrolled)	Once	Colonoscopy	58% (11/19) had clinical response Significant shift in fecal microbial diversity and composition toward donor’s profile Increased Treg cells (CD4+ CD25+ CD127lo) noticed in recipients’ lamina propria following FMT No serious adverse events recorded	[39]

Summary of some of the IBD clinical trials on the feasibility, safety and efficacy of FMT. Different study designs across varying degrees and types of IBD employing distinct techniques, volumes and frequencies of FMT administration, yielded different patients’ responses

*CD* Crohn’s disease, *UC* ulcerative colitis

### Application in UC

In a study of 7 children with ulcerative colitis, 4/7 responded to treatment 4 weeks post FMT administration based on their pediatric UC activity index. Detailed analysis indicated a shift of the class *Clostridia*, viromic and metabolomics profiles of respondents toward the donor’s microbiota composition. These changes alongside alterations in fecal concentrations of several other metabolites correlated with improved clinical remission [40]. A randomized clinical trial involving 73 adults divided participants into two groups of 38/73 (who received anaerobically prepared pooled donor FMT) and 35/73 (who received autologous FMT). At the eighth week post administration, 12/38 (32%) as against 3/35 (9%) of participants who received pooled donor FMT and autologous FMT respectively achieved primary end point remission. However only five of the 12 retained the clinical remission up to the 12th month [41]. Similarly, 9/36 (24%) patients who received FMT and 2/37 (5%) who received placebo attained clinical remission at 7 weeks post administration in another randomized controlled trial [28]. According to Paramsothy et al., specific bacteria and metabolites linked with the achievement of clinical therapeutic response in FMT include *Roseburia inulivorans* and *Eubacterium hallii*, and secondary bile acids and short-chain fatty acids respectively [42]. With respect to donor’s stool, the same clinical trial again reported that, enriched *Bacteroides* is associated with improved remission of patients while *Streptococcus species* is linked with no response to FMT [42].

### Application in CD

Increasing evidence indicates the promising outcome of FMT as an encouraging treatment option for Crohn’s disease (CD). However, the frequency of FMT for CD treatment remains unclear. In the first study that demonstrate that FMT could be a possible therapeutic option for CD, metagenomic analysis of stool microbiota indicated an evidence of FMT engraftment in 7/9 (78%) of participants. Analysis based on pediatric Crohn’s disease activity index showed 7/9 participants in clinical remission at 2 weeks and 5/9 patients who did not receive further medication in clinical remission at 6 and 12 weeks [43]. In another study, Vaughn et al. documented an encouraging clinical response rate of 11/19 (58%) in CD patients who received FMT treatment [39]. This was a consequence of a significant shift in patients’ fecal microbial diversity and composition towards their donor’s profile. Patients’ lamina propria also witnessed increased regulatory T-cells (CD4+ CD25+ CD127lo) following FMT treatment. A single FMT administration in children with active IBD including CD, resulted in a clinical response rate of 57% and 28% at 1 and 6 months post FMT respectively. Adverse events ranged from mild to moderate and self-limiting reactions [44]. This indicates that, although single FMT administration is safe in children, it is confronted with short-lived clinical response. In the quest to retain a long term remission in the first treatment in CD, Li et al. estimated the optimal timing for a second FMT administration. They concluded that, patients with CD could be given the second course of FMT in less than 4 months after the first FMT [45].

It is undoubtedly clear that the exploitation of the gut microbiome could produce a great therapeutic novelty. However, there is poor and limited knowledge on microbiota modulation in IBD, hence the necessity for further in-depth investigations. In order to achieve a reliable safety and efficacy assessment with solid conclusion on FMT application in IBD, it is essential to mount more randomized controlled trials. Again, the frequency of FMT administration, standardization of microbiome analysis and donor selection should also be intently investigated. Other optimal parameters of FMT effects with regard to route of administration, volume, preparation, and the type and disease severity should also be defined. Additionally, studies to assess the long-term FMT-mediated maintenance of clinical remission in CD and UC should be carried out.

## MSC therapy in IBD

The functions of MSCs spanning from cell replacement to immunosuppression and trophic factors production, have gain a lot of interest with increasing application in regenerative medicine and immune intervention in both experimental models and clinical trials. MSCs are known to interact with inflammatory cytokines and greatly influence both adaptive and innate immune systems via producing immunomodulatory particles that control the progression of inflammation by affecting cells like macrophages, T cells, NK cells, dendritic cells and B cells [46, 47]. By employing these properties, MSC application in IBD has been applied to achieve cellular replacement, immunosuppression and other trophic actions, together resulting in highly promising outcomes in clinical trials [48, 49] and experimental studies [50, 51]. MSC-therapy in IBD is mainly administered through systemic infusion or local inoculation. MSC extracts have also been applied in certain conditions. In one study that sought to overcome the low homing capacity of MSCs and augment their therapeutic influence, MSC extracts were used instead of whole cells in treating severe refractory IBD. Results indicated that the MSC extracts were highly potent than whole MSCs in reducing DAI, nitrite levels and the histological score. The extract totally inhibited the induction of inflammatory cytokines, recovered the destroyed epithelial barriers and shifted macrophage from M1 to M2 via reducing the levels of Chemokine (C–X–C motif) ligand 9 (CXCL9), monocyte chemoattractant protein-1 (MCP1) and inducible nitric oxide synthase (iNOS), but increasing the expression of Chemokine (C–C motif) ligand 1 (CCL1), IL-10, and Arg-1 [52]. Some clinical trials involving MSC-based therapy in IBD are summarized in Table 2.

**Table 2:** Application of MSC-based therapy in IBD clinical trials

IBD type	Study design	MSC source	Outcome	References
Moderate to severe UC	Phase I/II randomized controlled study	Human umbilical cord	30/36 patients showed good response with markedly improved mucosa at 1 month Decreased median Mayo score and histology score during follow up No evident adverse reactions after MSC infusion	[53]
CD	Randomized controlled study	Human umbilical cord	Decreased CDAI, HBI, and corticosteroid dosage with remarkable mucosal recovery at 12 months Concomitant anal fistula was improved in six patients treated with MSC	[54]
Luminal CD refractory to biologic therapy	Phase 2, open-label, multicenter study	Bone marrow	Improved recovery associated with reduced CDAI and CDEIS scores 7/15 patients had a clinical response, 8/15 had clinical remission, and 7/15 had endoscopic improvement	[55]
Complex perianal fistulas in CD	Phase 3 randomized double-blind controlled trial	Adipose	53/107 (50%) of MSC treated patients achieved combined remission in intention-to-treat protocol 53/103 (51%) of modified intention-to-treat populations achieved combined remission	[48]
UC	Two years observation after MSC treatment	Bone marrow	72.7% of UC patients who received MSC treatment achieved significant response Reduced activity of autoimmune inflammation and stimulated reparative process in the intestinal mucosa Increased duration of remission, reduced risk of recurrence of disease, and reduced frequency of hospitalizations	[56]
UC	–	Bone marrow	Increased in the duration of remission in patients with chronic recurrent and continuous recurrent course of UC Reduced risk of relapse, and reduced frequency of hospital admissions compared with medication therapy	[57]
Crohn’s perianal fistula	MSC safety study in pregnancy	Adipose	Fertility and pregnancy outcomes were not affected by MSC treatment No signs of treatment-related malformations were observed in the neonates by their respective pediatricians	[58]

Summary of some of the IBD clinical trials on the feasibility, safety and efficacy of MSC therapy. Different study designs across varying degrees and types of IBD employing distinct techniques and sources of MSC yielded different patients’ responses

*CDAI* Crohn’s disease activity index, *HBI* Harvey–Bradshaw index, *CDEIS* Crohn’s disease endoscopic index of severity

### Application in UC

Several clinical trial studies have investigated the safety and therapeutic influence of MSCs in UC. In one of such studies, the diffused and deep ulcers formed as well as severely inflamed mucosa of 30/36 (83%) participants were greatly improved at 1 month post MSC treatment [53]. Systematic review and meta-analysis of clinical and experimental studies was recently conducted on MSC-based therapy in UC. Out of the 15 studies included in the analysis, 7 were human (n = 216) trials and 8 were animal (n = 132) studies. The data showed that animals given MSCs had significantly lower DAI, longer colon length and lower histopathological score compared with control group. The clinical trials analysis also indicated an obvious recovery with single-arm studies analysis of four trials demonstrating an increased healing rate of 0.787 post-MSC treatment [59]. One of the challenges of MSC-based therapy is the issue of dosage and frequency of administration. In investigating the dose dependency of MSC therapy in colitis, Robinson et al. reported that, increasing doses above 1 × 10^6^ MSCs does not add additional therapeutic benefits than 1 × 10^6^ MSCs in preventing enteric neuropathy associated with intestinal inflammation [60]. Several other recent studies have also focused on enhancing the inherent therapeutic properties of MSCs to ensure consistency and efficacy in their application. For an example, the co-administration of the experimental drug MIS416 and human umbilical cord MSCs, exerted significant therapeutic efficiency consequently alleviating the symptoms of colitis as compared to the single MSC treatment [61]. The MIS416 was found to modulate the colon immune milieu via nucleotide-binding oligomerization domain-containing protein 2 (NOD2) and toll-like receptor 9 (TLR9) signaling activation, causing the MSCs to be readily recruited to the injury site to inhibit inflammation. Again, treatment of colitis with preconditioned MSCs resulted in an improved therapeutic effects characterized by increased body weight recovery, reduced DAI, reduced histological colitis score and decreased destruction of the epithelial barrier [62]. Further analysis revealed the activation of the extracellular signal-regulated kinases (ERK) pathway (inducing anti-apoptotic effects), suppression of T cell proliferation and inhibition of inflammatory cytokines TNFα and IL-2 whilst triggering the production of the anti-inflammatory cytokine IL-10 in T-cells.

### Application in CD

A randomized controlled clinical trial analysis reported that, umbilical cord derived MSCs were generally effective in the treatment of CD although it produced mild adverse events. At 12 months post-treatment, the Crohn’s Disease activity index (CDAI), Harvey–Bradshaw index (HBI) and corticosteroid dosage of the MSC-group, had reduced by 62.5 ± 23.2, 3.4 ± 1.2, and 4.2 ± 0.84 mg/day respectively as compared to the control group which had 23.6 ± 12.4, 1.2 ± 0.58, and 1.2 ± 0.35 mg/day reduction [54]. With regards to luminal CD, an open-label multicenter study involving 16 participants having CD refractory to biologic therapy was carried out. Among the 15 participants who completed the study, 12 (80%) had clinical response, 8 (53%) had clinical remission and 7 (47%) had endoscopic improvement [55]. The potent immunomodulatory effects exerted by MSCs during CD treatment is via complex paracrine and cell–cell contact mediated actions involving antigen-specific T cells [63].

Perianal CD occurs in approximately 25% of individuals with CD and is notoriously very difficult to treat with available biologics and surgical procedures. However, MSC therapy has shown encouraging outcomes. In their phase 3 randomized double-blind controlled trial, Panés et al. treated complex perianal fistulas in Crohn’s disease with allogeneic expanded adipose-derived MSCs. Results of intention-to-treat protocol indicated that, 53/107 (50%) of MSC treated patients achieved combined remission as against 36/105 (34%) of placebo treated patients. In modified intention-to-treat populations, MSC verses placebo resulted in 53/103 (51%) and 36/101 (36%) remission rates respectively [48]. Other documented evidence of MSC efficacy and safety in Crohn’s fistula include complete healing in 21/26 patients (80.8%) in modified per protocol analysis and 27/36 patients (75.0%) in modified intention-to-treat analysis [64] and 71% in a phase II clinical trial [65]. These among other studies have shown the efficacy and safety of MSC-based therapy in CD even in those that do not respond to conventional and/or biological treatments.

Despite the increasing trend in interest and significant clinical efficacy of MSC therapy in IBD, it is still confronted with unresolved challenges. Just as discussed in FMT, MSC therapy also has issues of administration protocol (route, dosage, schedule), origin and type of MSCs (autologous or allogeneic), quality of preparation and selection of experimental or clinical design to ensure optimum therapeutic impart. Conditions that potentiate the functions and desired effects of MSCs should further be investigated.

## Combined therapeutic effects

Fundamentally, the gut microbiota and host’s immune system inter-depend on each other by shaping the development, composition and functions of one another [66]. Invariably, MSC-therapy does not only aim at restoring the desired host immune response but also correct the altered gut microbiota whilst FMT restores gut dysbiosis resulting in dampened inflammation. These functions interlace and may even yield higher therapeutic influence when co-administered since the few existing investigations report the close communication and enhanced functionality of each other in their interactions [47, 67–70, 71].

The therapeutic imparts of MSCs do not necessarily rely on their full cellular engraftment, but rather on their capability to express trophic factors and hinder pathogenic immune reactions, favoring tissue repair [72, 73]. To this effect, conditioned-MSCs effectively alleviated colitis at both the inductive and recovery phases by producing these factors of anti-inflammation, proliferation and tissue remodeling [73]. It has also been demonstrated that, the microbiota intensely participate in the modulation of several host metabolic pathways, which cause the activation of immune-inflammatory axes and signaling pathways [74]. These functions among other desired therapeutic effects have been documented in the use of both FMT and MSC therapies in IBD.

**Fig. 2:**
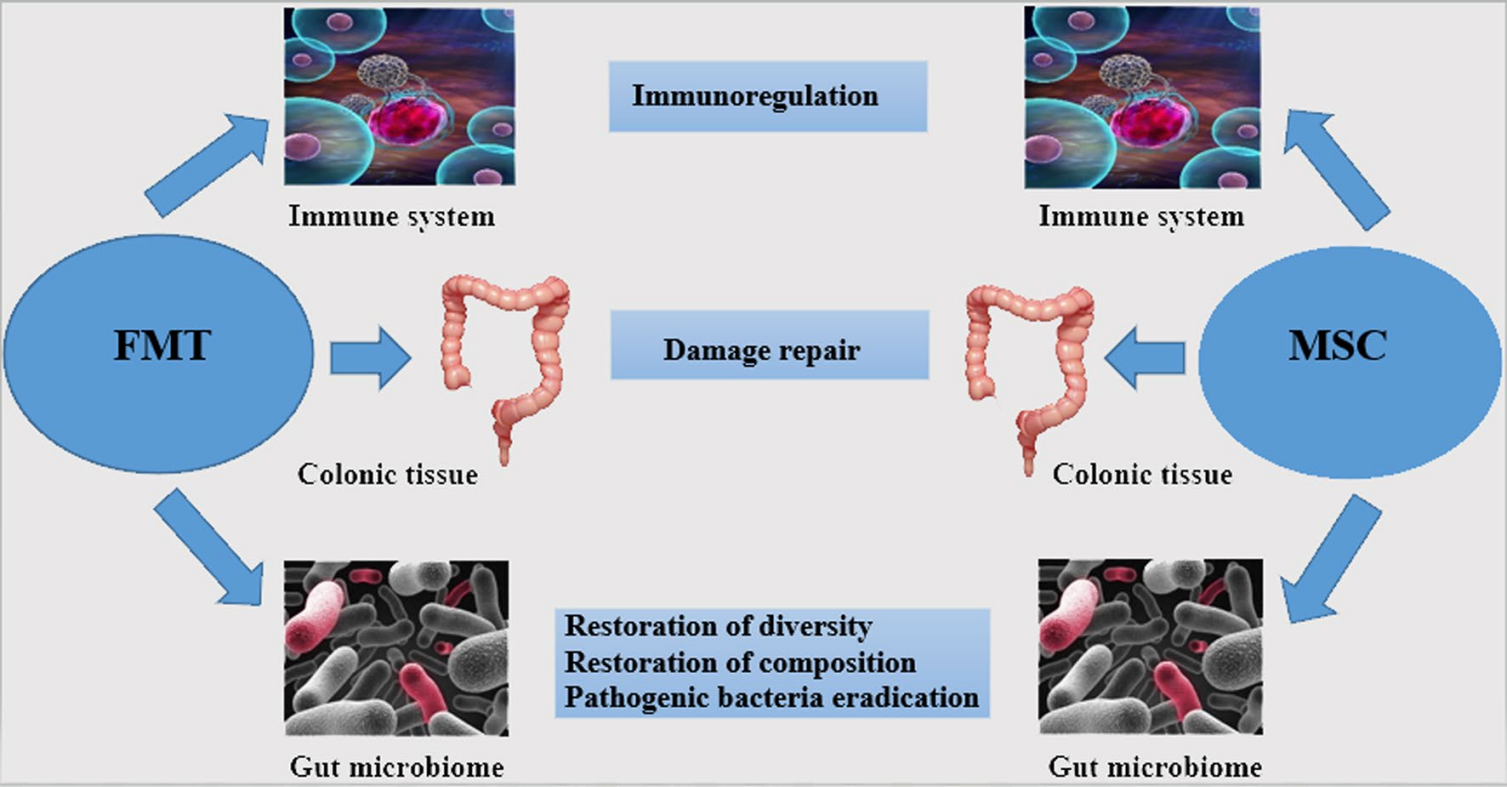
General therapeutic points of connection between FMT and MSC-therapy in IBD. The three main desired functional effects of the two transplants are immunoregulation aimed at dampening inflammation, tissue damage repair via proliferation and remodeling, and gut microbiota restoration including elimination of pathogenic bacteria

MSC–microbe interactions have a pronounced influence on the functions of MSC including its immunomodulation and migration, which are pivotal in the therapeutic utility of MSC across various diseases including IBD [70]. Known examples of the interaction between these two therapies are summarized in Table 3. Gastrointestinal bacteria are capable of inducing immune-regulatory mediator secretions, cytokine gene transcription and surface protein expressions in MSCs [70]. While Xiao et al. revealed that, microbiota alters the differentiation potentials and improves the immunomodulation ability of bone marrow MSCs [67], another research also indicated that a restored diversity of gut microbiome, reinstates bone marrow-derived MSCs from premature age-associated deterioration and loss of cell power of growth and division (senescence) [47]. Again, Nagashima et al. recently discovered a sub-epithelial mesenchymal cells which did not only induce gut microbiota diversity but also regulated the production of IgA which preserves gut symbiotic equilibrium [69]. Likewise, MSCs given by infusion caused an initial alteration in Bacteroidetes/Firmicutes ratio, which sustained intestinal mucosal function and homeostasis; believed to be valuable in hepatocyte repair [75]. Based on these findings, it could be hypothesized that FMT which seeks to restore gut microbial diversity and composition, may as well improve the functionality of MSCs and vice versa when co-administered in IBD treatment. This provides a promising area for future studies in IBD therapy. Figure 2 illustrates the main characteristic functional points of connection between the two therapies.

**Table 3:** The influence of gut–bacteria on the functions of MSCs

Gut bacteria	Source of MSC	Experimental condition	Pathways/secretomes involved	Outcome of interaction	References
Specific-pathogen-free (SPF) gut microbiota	Bone marrow	DSS-induced colitis	Cell metabolic, HIF-1/inflammatory signaling, and neurodegenerative pathways	Altered MSC differentiation potential Enhanced immunomodulation capacity of MSC Decreased disease activity index	[67]
*Lactobacillus acidophilus*	Canine adipose	In vitro	Increased transcription of key immunomodulatory genes, like COX2, IL6 and IL8 Significantly increased PGE2	Enhanced immunoregulatory function No induction of MSC death, degeneration or diminished proliferation No effect on MSC migration	[70]
*Salmonella typhi*	Canine adipose	In vitro	Increased transcription of key immunomodulatory genes like COX2, IL6 and IL8	No induction of antigen-presenting phenotype Increased capacity of MSCs to inhibit mitogen-induced T-cell proliferation Induction and expression of PPARγ, IL-6, IL8, HGF, COX2, CD54 and PGE2 Impeded MSC migration	[70]
Restored composition and diversity of gut microbiota with *Lactobacillus*	Bone marrow	Chronic hypoxic rats	–	Restored defect of senescence, poor cell proliferation, cell cycle arrest and multi-lineage differentiation deficiency in MSCs Reduced d-galactose accumulation	[47]
Lactobacillus rhamnosus GG	Lamina propria of the villus	Intestinal radioprotection in vitro	TLR2 and COX-2 dependent induction	*Lactobacillus rhamnosus* GG produced LTA, which then primed the epithelial stem cell niche to protect epithelial stem cells by activating macrophages and PGE2 secreting MSCs	[76]
*Helicobacter pylori*	Bone marrow	GIT infection by *H. pylori*	Over-expression of TNFα and CCL2 TNFα leads to activation of NF-κB-dependent pathway	Stimulated migration of MSC	[77]

This presents a sum-up of documented impacts of gut microbiome including some pathogenic bacteria on the functions of MSCs. In each demonstration, certain functions of MSCs were mostly improved with no distortion to the inherent properties

*PPARγ* peroxisome proliferator activator receptor gamma, *IL* interleukin, *HGF* hepatocyte growth factor, *COX2* cyclooxygenase 2, *PGE2* prostaglandin 2, *NF-κB* Nuclear Factor-kappa B, *TLR2* toll-like receptor 2, *LTA* lipoteichoic acid, *CCL2* C–C motive Chemokine ligand 2, *TNFα* tumor necrosis factor α

### Pathogenic bacteria eradication

The gut–microbiota is known to effectively contribute to host defense against pathogens by preventing their colonization [78], secreting direct target bacteriocin [79], antimicrobials peptides and RegIIIγ [80] and triggering immune cells [81]. For lack of or altered gut microbiome, germ-free and antibiotic treated mice are known to be extremely susceptible to enteric bacterial pathogens like *Listeria monocytogenes*, *Shigella flexneri, Salmonella Typhimurium* and *Citrobacter rodentium* [82, 83]. Gut microbiota mount host defense against pathogenic colonization by competitive nutrient utilization and by secreting antimicrobial factors like lactic acid, bactriocin and RegIIIγ [18] in addition to improving mucosal barrier functions and innate immunity [84]. On the other hand, it was demonstrated that MSCs significantly decreased bacteremia and mortality in sepsis partly by improving bacterial clearance and the phagocytic properties of blood monocytes [85], enhancing pathogen clearance [86] and by prostaglandin E2-dependent reprogramming of host macrophages which upregulates interleukin-10 production [87]. MSCs again, augmented the antibacterial function of neutrophil granules [88]. Harman et al. recently reported growth inhibition and cell membrane depolarization effects of equine derived/conditioned medium MSCs on *S. aureus* and *E. coli* with specific antimicrobial peptides [89] whilst Johnson et al. reported similar findings even in chronic drug-resistant bacterial infection [90]. Other antimicrobial secretomes of MSCs known to inhibit bacterial growth and/or kill them directly include cathelicidin [91], lipocalin 2 [92], elafin [89] and beta defensin 2 [93]. In the event of pathogen encounter, intestinal epithelial MSCs switch towards secretory epithelial cells differentiation [92], hence rapid proliferation and differentiation of goblet and Paneth cells. These cells produce resistin, mucin, trefoil factor 3 (TFF3), lysozyme and defensin to speed up bacterial clearance [94]. Toll-like receptors (TLRs) expressed on intestinal epithelial cells and mucosal dendritic cells (DCs) surfaces are known to be immune regulatory receptors and present bacterial antigens to the immune system [95], thereby differentiating commensal from pathogenic microbes. While TLR2, TLR5 and TLR4 identify extracellular microbes, TLR3 specifically recognizes viral particles with TLR4 playing a crucial function as a first protective line against probable pathogenic bacteria [96]. Both MSCs (by expression) [97, 98] and microbiome [99] are known to immunologically regulate the TLRs to enhance pathogenic eradication and stimulate anti-inflammation, even against antimicrobial resistant pathogens [80].

### Anti-inflammation

It has been shown that gut–microbiota induces the differentiation and expansion of colonic Regulatory T-cells (Tregs) [100] and the development of Th17 cells [101], both of which play roles in the regulation or suppression of other immune system cells. *F. prausnitzii,* was found to exert anti-inflammatory effect via producing IL-10 and inhibiting the secretion of inflammatory cytokines like IL-12 and IFN-γ [102]. The same gut bacterial is again associated with the release of anti-inflammatory molecules like salicylic acid within the gut lumen [103]. This anti-inflammatory effect is exerted on both immune cells and intestinal epithelial cells via specifically stimulating a new type of human IL-10 producing Treg cells [104] and bacterial-derived peptide inhibition of NFkB activation [105] respectively.

MSCs also trigger the upregulation of several anti-regulatory modulators such as Foxp3+ regulatory T cells, Th17 and Th1 cells in CD and Th2 cells in UC [106, 107]. They again upregulate Treg-cells, IL-10 and TGF-β whilst decreasing IL-17 [108]. Other studies have also reported MSCs to increase the secretion of pro-inflammatory cytokines Th1 and Th17 while downregulating inflammatory cytokines IFN-γ, TNF-α, IL-6, IL-2 and IL-17 [109, 110]. According to Ahmed et al., genetic expression of inflammatory markers (IL-23, IFN-γ, TNF-α, ICAM-1) within the intestinal mucosa of MSC treated mice appreciably lowered, resulting in a significant improvement in weight gain, stool condition, as well as normal histopathology of tissues analyzed [111].

**Fig. 3:**
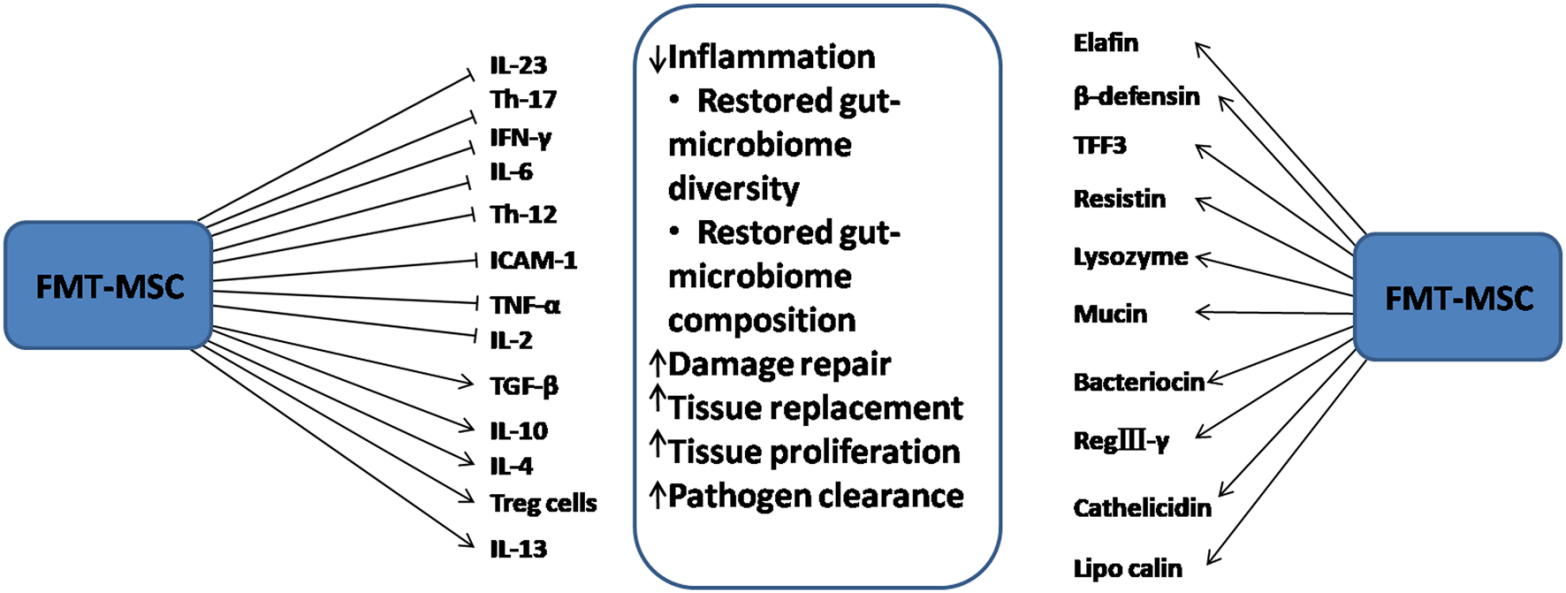
The combined effects of FMT–MSC secretomes in attenuating IBD. Several immunomodulatory factors are found in the inflammatory environment. With the quest to attenuate IBD, the administered MSCs and FMT regulate these modulators to inhibit inflammation and restore gut function. In the event of pathogenic bacterial colonization, a number of bactericides are expressed to eradicate infection

Kol et al. reported that, while particular in vitro manipulations could produce an antigen presenting cell (APC) phenotypic shift in MSCs, the communication with physiologically important bacteria including even pathogenic bacteria *Salmonella Typhi*, did not trigger this potential harmful phenotypic shift [70]. Further investigations indicated that, intestinal bacteria-MSCs interactions triggered the enzymatic precursor for PGE2 (i.e., COX2) and pleiotropic cytokine IL6, which are both anti-inflammatory and pro-inflammatory mediators that inhibit Th17 differentiation, lymphocyte proliferation and M1 differentiation of monocytes [70, 112]. The various secretomes elicited within the MSC and FMT administered environment and their resultant effects are shown in Fig. 3.

### Proliferation and tissue remodeling

Regardless of the source of MSCs and the route of administration, they have been shown to be capable of engrafting into inflamed intestinal and mesenteric lymph nodes in IBD and self-proliferating as well as inducing the proliferation of intestinal cells to repair and replace damaged tissues. By self-proliferation, MSCs trigger colonic repair by differentiating into several cells and dampening inflammation [113] and by differentiating into fibroblast [51]. Many other researches have indicated the ability of MSC to regenerate, remold and induce proliferation of tissues [114, 115]. For instance, a systemic infusion of MSCs improved the proliferation and differentiation of intestinal epithelial cells [114, 116] while MSCs-conditioned medium (MSC-CM) also strongly induced cell proliferation, tissue remodeling and repair of intestinal submucosa injury in colitis [73]. MSCs within the intestine direct epithelial cells lineage differentiation [117, 118] and secrete RANKL (TNFSF11), which is known to induce the development, differentiation and maintenance of M cells [69, 119, 120].

Gut microbiota constitute a complex ecosystem within which a progressive cross communication modulates several host cellular activities as well as metabolic pathways, including actively shaping and remodeling the mucosa of intestinal tissues [70]. Through NOD2 sensors, the gut–microbiota is associated with gut epithelial cell regeneration [121]. Although little is known about microbiome–MSCs interactions, certain researches have already documented the close communication between microbiota (and their products) and stem cells in the intestinal niche; potentially in the crypt, assisting stem cells in their roles of epithelial regeneration and homeostasis post-damage repair [121, 122]. In one of such investigations, a common peptidoglycan motif to all bacteria, triggered stem cell survival, leading to a strong cyto-protection against oxidative stress-mediated cell death [121]. Soontararak et al. reported that, the administration of induced pluripotent stem cells (earlier proven to be functionally equal to adipose derived MSCs), did not only trigger increase in Lgr5+ intestinal stem cells, but also upsurge the intestinal epithelial cells proliferation, angiogenesis and even significantly restored alterations in the gut microbiome of IBD mice [68].

## Discussions and future perspective

Currently, many preliminary investigations have demonstrated the therapeutic potentials of FMT in IBD [29] and gastrointestinal disorders [123, 124], among few other conditions. The outcome alongside reviews and meta-analysis have asserted that, FMT application in IBD is promising, effective and safe. However, it is confronted with challenges including minor to serious adverse events, unknown long-term stability of remission, low overall quality of available studies, undefined study designs and treatment protocols, donor and recipient factors as well as poorly defined efficacy endpoint. Again, the low remission rates witnessed in FMT application in IBD studies could be attributed to the complex interplay between microbial, environmental [125], genetic and immunologic [28, 126] factors that take part in the pathogenesis of IBD, therefore the introduction of just a new set of gut microbiota may not necessarily produce the expected outcome. Similarly, MSC therapy is confronted with similar challenges in its application in IBD in spite of the successes witnessed. While these challenges are being battled out in the mission to improve their therapeutic efficacy, there is the need to mount more investigations bent on throwing more light on microbiome–MSC interactions. Although it is asserted that, MSC–microbiome communications occur via TLRs [98] and NLRs [127], the direct and specific proof of such contacts and their resulting impact on the immunomodulatory capability of MSCs remain undefined. Gut microbiome along with other modulators may even contribute to establishing engrafted MSC’s niche in IBD during MSC therapy, and determine whether the given MSCs will take on a pro-inflammatory or an anti-inflammatory phenotype [128, 129]. More researches focused in this area may result in the discovery of a novel product/mechanism of their communication in therapy, since the few available data appear highly encouraging.

Although it is not certain whether it is the MSC-activated effects that enhance the microbiome diversity or rather the opposite, by and large, both effects within the colon epithelium improve each other’s functions, consequently encouraging intestinal epithelial cells regeneration, dampened inflammation, pathogen eradication and angiogenesis. While FMT and MSC therapies are confronted with several challenges in their utility in IBD, a combined therapeutic approach may successfully yield an increased clinical response and remission since both therapies do not only share common characteristics in impart but also influence each other to enhance and potentiate their functionality and therapeutic efficacy. Also, the identification and subsequent administration of only the specific gut–bacteria responsible for eliciting desired effects in IBD treatment, would yield better outcome than the administration of the bulk fecal material. Moreover, more studies geared toward elucidating not only host–microbiome interactions but also cross-microbiome interactions are expected to improve FMT. Finally, for a prospective utility of an FMT–MSC combined therapy in IBD and other conditions, there is the need to further investigate the interaction between the components of these two therapies and clearly understand the mechanisms underlying the enhanced effects on each other.

Considering the increasing interest on these two therapies and the highly promising outcome of MSC–gut microbiota communication in the few available documents, this field would soon attract more investigations and applications across many conditions with significant daily medical practice impact.

## Conclusion

The participation of gut–microbiota in the pathophysiology of IBD is well established. The gut–microbiota provide several health benefits to the host including pathogen protection, cellular regeneration and immune modulation. With the background that, alterations in the function and composition of the gut–microbiota, coupled with immune-dysregulation lead to chronic and relapsing intestinal mucosa inflammation (i.e. IBD), the application of MSCs and FMT as therapies in IBD have gain much interest. These two therapies seek to resolve the underlying dysbiosis and repair damages. Irrespective of the successes, increasing trend in interest and the significant clinical efficacy of both therapies in IBD, they are still confronted with several unresolved challenges. In the phase of these challenges, stem cells–gut microbiota interaction is fast emerging as a novel and highly promising field. In the communication between MSCs and gut microbiota, the functions of each component are improved; in that while MSC reinstates gut microbiota composition and diversity, FMT also potentiates MSC activities. This could lead to higher clinical remission rates when applied together in the IBD environment, hence the need to explore further.

## Data Availability

Not applicable.

## Abbreviations

IBD: inflammatory bowel disease
MSCs: mesenchymal stem cells
FMT: fecal microbiota transplantation
CD: Crohn’s disease
UC: ulcerative colitis
NK cells: natural killer cells
DAI: disease activity index
CXCL: Chemokine (C–X–C motif) ligand
MCP: monocyte chemoattractant protein
iNOS: inducible nitric oxide synthase
CCL: Chemokine (C–C motif) ligand
IL: interleukin
NOD2: nucleotide-binding oligomerization domain-containing protein 2
TLR: toll-like receptor
ERK: extracellular signal-regulated kinases
TNF: tumor necrosis factor
HBI: Harvey–Bradshaw index
CDAI: Crohn’s disease activity index
CDEIS: Crohn’s disease endoscopic index of severity
PPARγ: peroxisome proliferator activator receptor gamma
HGF: hepatocyte growth factor
COX2: cyclooxygenase 2
PGE: prostaglandin
NF-κB: nuclear factor-kappa B
LTA: lipoteichoic acid
TFF3: trefoil factor 3
DC: dendritic cell
Th cells: T-helper cells
Tregs: regulatory T-cells
IFN: interferon
ICAM: intercellular adhesion molecule
APC: antigen presenting cell
